# Hyperspectral imaging of common foodborne pathogens for rapid identification and differentiation

**DOI:** 10.1002/fsn3.1131

**Published:** 2019-07-10

**Authors:** Minto Michael, Randall K. Phebus, Jayendra Amamcharla

**Affiliations:** ^1^ School of Food Science Washington State University Pullman WA USA; ^2^ Food Science Institute Kansas State University Manhattan KS USA

**Keywords:** hyperspectral imaging, pathogens, rapid identification

## Abstract

Hyperspectral imaging (HSI) provides both spatial and spectral information of a sample by combining imaging with spectroscopy. The objective of this study was to generate hyperspectral graphs of common foodborne pathogens and to develop and validate prediction models for the classification of these pathogens. Four strains of *Cronobacter sakazakii*, five strains of *Salmonella* spp., eight strains of *Escherichia coli*, and one strain each of *Listeria monocytogenes* and *Staphylococcus aureus* were used in the study. Principal component analysis and *k*NN (*k*‐nearest neighbor) classifier model were used for the classification of hyperspectra of various bacterial cells, which were then validated using the cross‐validation technique. Classification accuracy of various strains within genera including *C. sakazakii*, *Salmonella* spp., and *E. coli*, respectively, was 100%; except within *C. sakazakii*, strain BAA‐894, and *E. coli*, strains O26, O45, and O121 had 66.67% accuracy. When all strains were studied together (irrespective of their genus) for the classification, only *C. sakazakii* P1, *E. coli* O104, O111, and O145, *S*. Montevideo, and *L. monocytogenes* had 100% classification accuracy, whereas *E. coli* O45 and *S*. Tennessee were not classified (classification accuracy of 0%). Lauric arginate treatment of *C. sakazakii* BAA‐894, *E. coli* O157, *S*. Senftenberg, *L. monocytogenes*, and *S. aureus* significantly affected their hyperspectral signatures, and treated cells could be differentiated from the healthy, nontreated cells.

## 1 INTRODUCTION

Hyperspectral imaging (HSI) is an emerging technology that has a great potential in rapid detection and identification of foodborne pathogens. The HSI was originally designed for remote sensing and now has found its application in various fields such as astronomy, agriculture, pharmaceutical, and medicine (Gowen, O'Donnell, Cullen, Downey, & Frias, 2007; Smith, 2012). The increased food safety and defense concerns due to foodborne pathogens have reinforced the need for rapid pathogen detection and identification methods in food. Although traditional methods are still used for the detection and identification of pathogens in food, these methods are cumbersome, labor‐intensive, expensive, and can take from 4 to 7 days to give confirmatory results. Therefore, rapid detection methods for foodborne pathogens, at least at the presumptive level, are required for the functioning of a safe and fast food supply chain.

The HSI is a nondestructive method of analyzing and detecting a specimen and combines imaging with spectroscopy to acquire both spatial and spectral information of a specimen (such as bacterial cells or colonies) by using visible near‐infrared spectra (400 to 1,000 nm; Dale et al., 2013; Gowen et al., 2007). The HSI utilizes optical characteristics of specimens for the identification; therefore, HSI works on the interactions between light (visible and/or near‐infrared light) and the molecular structure of the specimen. During HSI, hyperspectral images of a sample are captured at various predefined wavelengths; and reflectance, transmittance, absorbance, or fluorescence (depending on the molecular and chemical structure of a sample) are measured over this wavelength range to generate hyperspectral graphs. These hyperspectral graphs, used to develop a library for different organisms, can then be used for the rapid identification of an unknown sample.

Rapid and early detection of pathogens in food is vital at every stage of processing, especially for “zero tolerance” pathogens such as *Escherichia coli* O157:H7 and Big Six Shiga toxin‐producing *E. coli* (STEC; O26, O45, O103, O111, O121, and O145) in ground beef, and *Listeria monocytogenes* in ready‐to‐eat foods (Buchholz & Mascola, 2001; FSIS, 2012a; 2012b). Another sensitive area where early and faster detection of pathogens is vital is the detection of *Salmonella* spp. and *Cronobacter sakazakii* in powdered infant formula manufactured for newborns and infants.

The previous research done utilizing HSI for the rapid identification and differentiation of various pathogens has been conducted on a macro‐scale using bacterial colonies grown on a media agar, but best to our knowledge, studies at microscale (at the single bacterial cell level) are scarce in the literature (Eady & Park, 2016). Using HSI, Yoon et al., (2013a), Yoon et al. (2013b) were able to identify Big Six STEC grown on Rainbow agar as pure and mix cultures at accuracy levels from 80% to 100%. Fiore, Casale, and Aureli. (2008) were able to discriminate maize kernels infected with *Aspergillus niger* and *A*. *flavus* from uninfected kernels using HSI. The U.S. Department of Agriculture's Agriculture Research Service (ARS) developed and successfully tried (under commercial conditions) a HSI system to detect and separate fecal contaminated poultry carcasses from the good carcasses (FSIS, 2013). Factors like morphology of colonies grown on media agar and presence of food components on food surfaces, along with individual or clusters of bacterial cells, can interfere with HSI and affect the hyperspectra. Therefore, HSI of bacteria at the cellular level should be studied to exclude interfering factors in generating hyperspectra of targeted bacteria.

It is also very relevant and important to study whether inactive or injured bacterial cells can be differentiated from active and live bacterial cells using HSI (Anderson, Reynolds, Ringelberg, Edwards, & Floey, 2008). Lauric arginate (LAE) is a generally recognized as safe (GRAS) food‐grade antimicrobial exhibiting activity against a wide range of pathogens (Saini, Miguel, Marsden, Getty, & Fung, 2013). The LAE has been studied for its use in food as an antimicrobial treatment and as a sanitizer for food contact surfaces (Becerril, Manso, Nerin, & Gómez‐Lus, 2013; Saini, 2012). The LAE disrupts the plasma membrane of bacterial cells, which alters the metabolic processes and inhibits normal cellular cycles (Bakal & Diaz, 2005). Therefore, it is essential to study the effect of antimicrobials, such as LAE, on bacterial cells and subsequently on the HSI of these cells treated with antimicrobials.

Therefore, the main objective of this research was to study whether hyperspectral graphs/signatures obtained from HSI of individual bacterial cells could be used for the rapid presumptive identification and differentiation of various strains of pathogenic bacteria. This included the development and validation of classification models for common foodborne pathogens. The effect of antimicrobial (LAE) treatment of various strains on hyperspectral signatures of these bacteria was also studied.

## 2 MATERIALS AND METHODS

### 2.1 Experimental design

To study whether HSI can be used for the rapid identification and differentiation of various foodborne pathogens, four strains of *C. sakazakii*, five strains of *Salmonella* spp., eight strains of *E. coli*, and one strain each of *L. monocytogenes* and *S. aureus* were used in the study. All strains were individually grown on tryptic soy agar (TSA; Difco, Becton Dickinson Company) for isolation, and hyperspectral images of these cells from the isolated colonies were captured. These images were then used to generate hyperspectral graphs of respective bacterial cells that were stored in a reference library. Principal component analysis (PCA) and *k*NN (*k* Near Neighbor) classifier model were used for the classification of hyperspectra of various pathogens, followed by the “cross‐validation” technique. For LAE treatment, one strain from each genus was grown in TSB for 24 hr and then treated with 2,000 ppm LAE followed by HSI. Three replications were conducted for each strain for generating hyperspectral graphs; within each replication, HSI of various strains was conducted randomly.

### 2.2 Culture propagation

The seventeen Gram‐negative (four *C. sakazakii*, five *Salmonella* spp., and eight Shiga toxin‐producing *E. coli*) and two Gram‐positive (*L. monocytogenes* and *S. aureus*) foodborne pathogenic bacteria used in this study are presented in Table 1. All strains were selected on the basis of risk and involvement in foodborne disease outbreaks, or isolated from the environment and food processing facilities. All cultures were stored on TSA at 4°C and confirmed using API^®^ 20E (for *C. sakazakii, Salmonella* spp., and *E. coli* strains; Biomérieux), API^®^ *Lister* (for *L. monocytogenes*), and API^®^ Staph (for *S. aureus*).

**Table 1:** Gram‐positive and Gram‐negative bacteria used in the study

Bacteria	Strain/Serotype	Source
*Cronobacter sakazakii*	BAA−894	ATCC^a^
	Environmental isolate (E1)	University of Nebraska, Lincoln
	Processing plant isolate 1 (P1)	University of Nebraska, Lincoln
	Processing plant isolate 2 (P2)	University of Nebraska, Lincoln
*Salmonella* spp.	Agona BAA−707	ATCC
	Tennessee 10,722	ATCC
	Typhimurium 13,311	ATCC
	Montevideo	Kansas State University
	Senftenberg	Kansas State University
*Escherichia coli*	O26	University of Nebraska, Lincoln
	O45	University of Nebraska, Lincoln
	O103	University of Nebraska, Lincoln
	O104	University of Nebraska, Lincoln
	O111	University of Nebraska, Lincoln
	O121	University of Nebraska, Lincoln
	O145	University of Nebraska, Lincoln
	O157	Kansas State University
*Listeria monocytogenes*	SLR 2249	ATCC
*Staphylococcus aureus*	25923	ATCC

a American type culture collection

### 2.3 Bacterial cell samples preparation and immobilization

A loop (1 μl) from stock cultures stored on TSA was individually transferred into 10 ml TSB and incubated for 24 hr at 35°C for *L. monocytogenes* and *E*. *coli*, and 37°C for *C. sakazakii*, *Salmonella* spp., and *S. aureus*. Incubated strains were then individually streaked on TSA for isolation and incubated for 24 hr at respective temperatures. For each strain, one loop from an isolated colony was mixed in 1 ml filtered (0.2 μm) sterilized HPLC grade water (Fisher Scientific) in a microcentrifuge tube by vortexing for ~1 min. Ten μl of vortexed samples was individually transferred on clean and sanitized 1‐mm glass slides (Fisherfinest^®^, Fisher Scientific), and immobilized by air drying in a biosafety cabinet (SterilGARD^®^ II, The Baker Company) for ~5 min. These immobilized bacterial cells on glass slides were then used for the HSI analysis.

### 2.4 Lauric arginate treatment

For LAE (as Ethyl Lauroyl Arginate HCL; Vedeqsa Inc.) treatment, one strain from each genus was selected: *C. sakazakii* BAA‐894, *E. coli* O157:H7, *L. monocytogenes*, *S. aureus*, and *Salmonella* Senftenberg. According to the Food Safety and Inspection Service (FSIS) of the U.S. Department of Agriculture, LAE can be used in various meat and poultry products up to 200 ppm by weight of the finished product (FSIS, 2013). Based on the previous research conducted on LAE treatment of veal carcasses in our laboratory, in which ~2,000 ppm LAE solution was used to achieve a final 200 ppm LAE by weight of beef cut being treated, 2,000 ppm LAE concentration was selected for this study. A loop from the stock culture of all bacteria on TSA was individually transferred into 10 ml of TSB and incubated for 24 hr at respective temperatures. For all strains, a loop from the inoculated TSB was then transferred to 10 ml TSB tubes and again incubated for 24 hr at respective temperatures. For each strain, after inoculation, 3 ml of inoculated TSB was mixed with 6 ml of noninoculated TSB and 1 ml of LAE (to achieve the final concentration of 2,000 ppm of LAE) and stored for 5 min at 4°C. After LAE treatment, 5 ml of D/E Neutralizing Broth was immediately added to the tubes (to neutralize the solution and arrest the antimicrobial effect of LAE). Tubes were then centrifuged at 4,960 × *g* at −4°C for 15 min, supernatant was discarded, and pellets were washed with 10 ml 0.1% peptone solution and centrifuged again. After centrifugation, supernatant was discarded, and one loop from the pellet in centrifuge tube was used for HSI analysis of each strain.

### 2.5 Hyperspectral graphs generation

**Figure 1:**
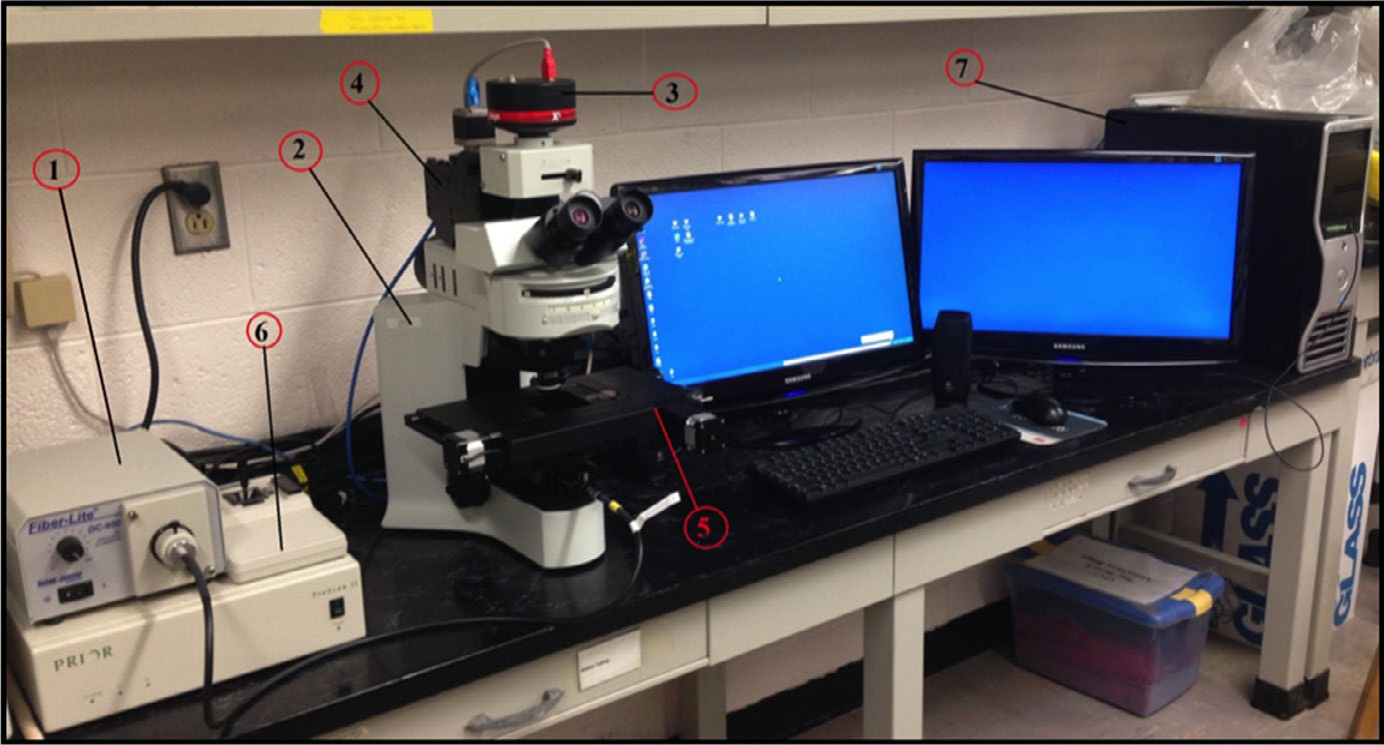
CytoViva^®^ Hyperspectral Imaging Microscope System. 1: Halogen light source; 2: optical microscope; 3: optical camera; 4: VNIR spectrophotometer; 5: motorized stage; 6: controller for stage; and 7: computer

A CytoViva^®^ microscope system (CytoViva, Inc.) used for capturing hyperspectral images in the study is presented in Figure 1. Environment for Visualizing Images (ENVI, Exelis Visual Information Solutions) software version 4.4 was used for analyzing acquired hyperspectral images and generating hyperspectral graphs. Hyperspectral images of individual bacterial cells on air‐dried glass slides were acquired by focusing the microscope at 4,000 × magnification and ENVI settings at: 0.5 s exposure time, low grain, and low spatial and spectral resolutions (as recommended by CytoViva^®^ personal communication). Hyperspectral images were acquired using “push broom” (also known as “line scan”) technique in which the glass slide supporting the dried culture on the motorized stage of microscope was moved across the field of view of the microscope during image acquisition. Using ENVI, three bacterial cells were selected from the acquired images as regions of interest (ROI). Average scattering values at respective wavelengths of these three ROI were used to generate hyperspectral graphs at wavelengths ranging from 400 to 1,000 nm (at wavelength interval of ~1.29 nm resulting in 465 wavelength bands). These graphs were then stored in a reference library for future use and comparison/classification.

### 2.6 Principle component analysis, and classification and validation of hyperspectral graphs

Preprocessing is the first important step in statistical analyses and classification of a spectral data set; however, no general guidelines or rules exist for selecting a particular preprocessing technique for a specific type of a data set (Scott, James, & Ali, 2007). The type of preprocessing technique used for a particular data set should aim to provide the best possible classification accuracy. For this study, spectral data/graphs were first preprocessed by reducing the number of wavelength bands from 465 (ranging from 400 to 1,000 nm) to 255 (ranging from 425.57 to 753.84 nm). Hyperspectral graphs were then preprocessed by normalizing the *y*‐axis (scattering value) from values 0 to 1 (Scott et al., 2007), with “1” being the brightest point on the ROI and “0” being the darkest point. The following equation was used to calculate normalized scattering values (Scott et al., 2007):

$$X_{\mathrm{ij}}=\left[X_{\mathrm{ij}}-\text{min}\left(X_{j}\right)\right]/\left[\text{max}\left(X_{j}\right)-\text{min}\left(X_{j}\right)\right]$$

where, *X_ij_* is normalized scattering value at a particular wavelength; *X_ij_* is actual scattering value at a particular wavelength; min (*X_j_*) is minimum scattering value; max (*X_j_*) is maximum scattering value.

Unsupervised PCA of normalized hyperspectral graphs was conducted to reduce the dimensionality and redundancy in the data set (scattering values at the respective wavelengths) for the classification (Jun, Kim, Lee, Millner, & Chao, 2009). The new coordinates of the spectral data with reduced dimensions are called principal components (PC), which explain the variability within the data (Del Fiore et al., 2010). The PCs explaining 99% of the variability in the data set were used for the further analysis and classification of hyperspectral graphs. Principal component analysis plots for various sets of hyperspectral graphs were plotted using the first two PCs for approximate visualization of differences and similarities among the strains. The mathematical model was constructed using the following equation (Romía & Bernàrdez, 2009):

$$X\;=\;{\text{TP}}^{T}+\;E$$

where, **X** (**K** × **N**) data set matrix is reduced to a much smaller number of A variables called principal components (PC), **T** (**N** × **A**) is a matrix containing A scores for PCs, **P** (**K** × **A**) that containing the A loadings for the PCs, and **E** (**K** × **A**) the residual matrix of the model.

The *k*NN classifier modeling technique was used for the classification of different sets of strains, where *k* value was kept at 3. The *k*NN is a supervised classification technique used most commonly for data sets with little prior knowledge of data distribution. For the *k*NN classification, the training model requires sorting the training data, and the distances of samples from the training set with respect to the unclassified sample are calculated, and the unclassified sample is assigned to the group of the nearest *k* neighbors (Scott et al., 2007). In the kNN classification model, “*k*” is referred to as the number of close neighbors or samples to be considered for the grouping of the unclassified sample. The value of *“k*” can be any positive numeric value; however, values from 1 to 3 are commonly used (Scott et al., 2007; Yoon et al., 2013a; 2013b). These classification models were then validated using the cross‐validation technique.

## 3 RESULTS AND DISCUSSION

**Figure 2:**
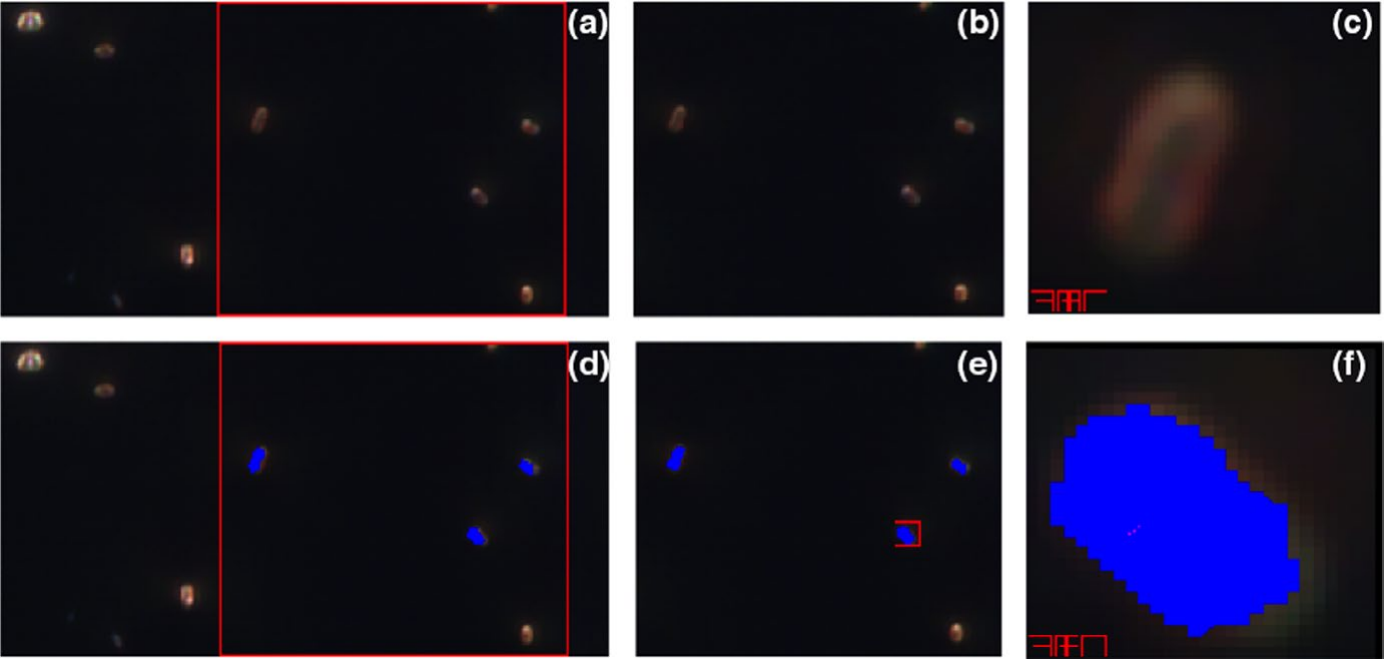
Hyperspectral Images of *Listeria monocytogenes* at 4,000 × magnification. a and d: Images of nonselected and selected regions of interest (ROI) as appears under the field of view of microscope, respectively; b and e: images of nonselected and selected ROI at first zoom, respectively; and c and f: images of nonselected and selected ROI at second zoom, respectively

An example of the image of bacterial cells as visible under the field of view of the microscope and acquired by the CytoViva^®^ microscope system is presented in Figure 2a. Using ENVI software, this view was further zoomed at two levels as presented in Figure 2b,c. The appearance of bacterial cells at different zoom levels after the ROI was selected for acquiring hyperspectral graphs is presented in Figure 2d–f. The average time required to acquire images was ~5 min. The captured images were clarified using various image‐clarifying tools available in ENVI for the better visualization of bacterial cells; however, clarification of images did not affect the hyperspectral signatures of bacterial cells or other pixels in the images. Based on the preliminary work and personal communication with CytoViva^®^ personnel, wavelength range from 425.57 to 753.84 nm was selected for PCA and *k*NN classifier modeling because wavelengths below 425.57 nm and above 753.84 nm were overlapping for all bacterial strains and no useful information could be utilized for the differentiation and classification purposes.

**Figure 3:**
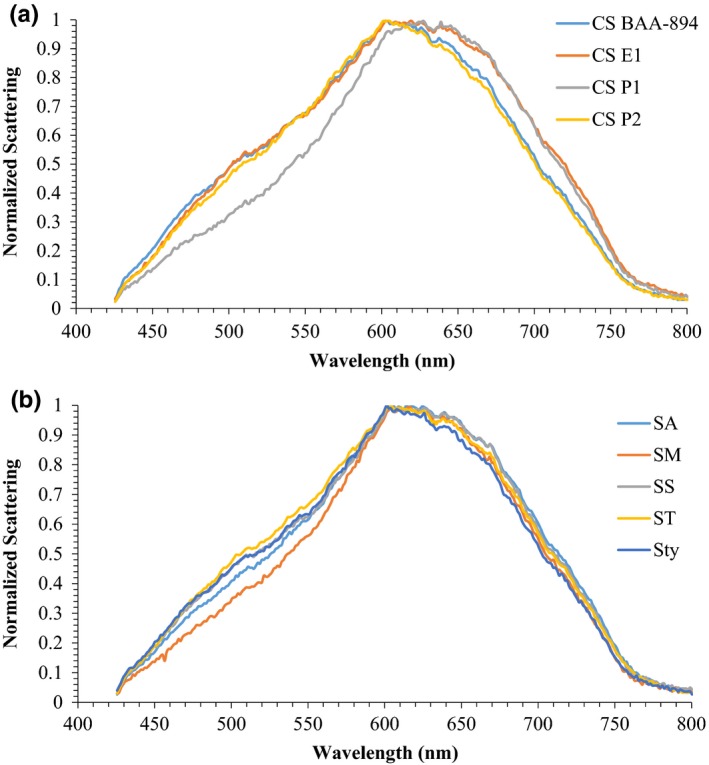
Hyperspectral graphs of: a) *Cronobacter sakazakii* (CS) BAA‐894, environment isolate (E1), and two food processing plant isolates (P1 and P2); b) *Salmonella* Agona BAA‐707 (SA), Montevideo (SM), Senftenberg (SS), Tennessee 10722 (ST), and Typhimurium 13311 (STy)

**Figure 4:**
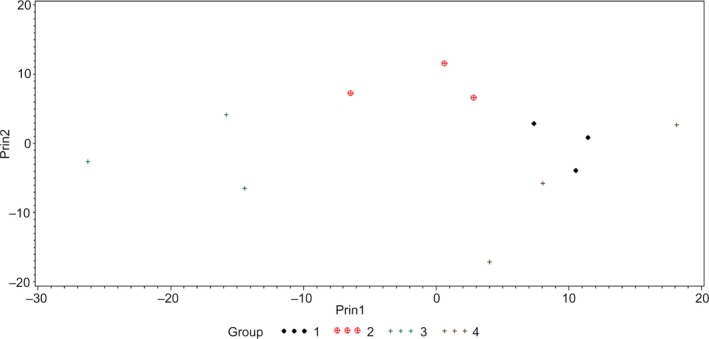
Principal component analysis (PCA) plot showing first two principal components (Prin1 and Prin2) of hyperspectral graphs of *Cronobacter sakazakii* BAA‐894 (1), environment isolate (2), and two food processing plant isolates (3 and 4)

To study the variation of hyperspectral graphs/signatures within a genus, HSI of different strains of *C. sakazakii*, *E. coli*, and *Salmonella* spp. was conducted. Classification models of these strains developed using PCA and *k*NN classifier were cross‐validated. Hyperspectral graphs of various strains of *C. sakazakii* are presented in Figure 3a. The graph indicates that *C. sakazakii* P1 had a different scattering pattern (shape of the curve) at wavelengths <615 nm compared with the other strains; however, at wavelengths >615 nm, the scattering patron of *C. sakazakii* P1 was similar to that of *C. sakazakii* E1. Although *C. sakazakii* BAA‐894 and P2 had different intensities, the scattering pattern of both strains was similar throughout the wavelength range (Figure 3a). The PCA plot of *C. sakazakii* strains shows that *C. sakazakii* P1 was grouped on the negative (left) side of the PC1, while *C. sakazakii* E1 was grouped around the center, and *C. sakazakii* 894 and P2 were grouped on the positive (right) side of PC2 (Figure 4). Classification accuracy of *C. sakazakii* E1, P1, and P2 was 100%; however, *C. sakazakii* BAA‐894 had classification accuracy of 66.67% (where *C. sakazakii* BAA‐894 was misclassified as *C. sakazakii* P2 at 33.33%; Table 2). These results suggested that hyperspectral signatures of *C. sakazakii* strains were different from each other, and HSI could be used for the rapid presumptive identification and differentiation of various *C. sakazakii* strains studied in this research, except that *C. sakazakii* BAA‐894 had 33.33% chance of being misclassified as *C. sakazakii* P2.

**Table 2:** Classification accuracy of various strains of *Cronobacter sakazakii*, *Salmonella* spp., and *Escherichia coli* within the respective genus obtained from cross‐validation of principal component analysis and *k*NN (k‐nearest neighbor, *k* = 3) classification*

Bacteria	Strain/Serotype	% Classification Accuracy	Misclassified As (%)
*Cronobacter sakazakii*	BAA−894	66.67	CS‐P2 (33.33)
	E1	100	–
	P1	100	–
	P2	100	–
*Salmonella* spp.	SA	100	–
	SM	100	–
	SS	100	–
	ST	100	–
	STy	100	–
*Escherichia coli*	O26	66.67	EC‐O103 (33.33)
	O45	66.67	EC‐O121 (33.33)
	O103	100	–
	O104	100	–
	O111	100	–
	O121	66.67	EC‐O45 (33.33)
	O145	100	–
	O157	100	–

**Figure 5:**
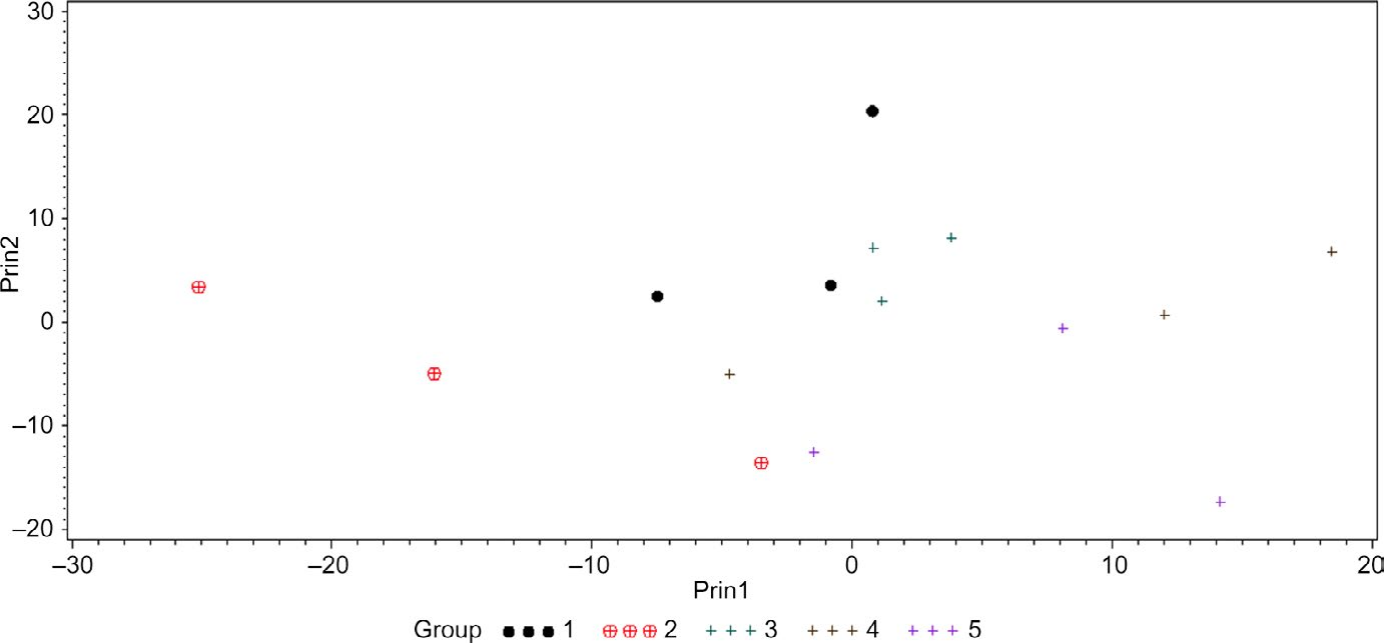
Principal component analysis (PCA) plot showing first two principal components (Prin1 and Prin2) of hyperspectral graphs of *Salmonella* Agona BAA‐707 (1), Montevideo (2), Senftenberg (3), Tennessee 10722 (4), and Typhimurium 13311 (5)

Within *Salmonella* spp., at wavelengths <600 nm, *S*. Montevideo had a different scattering pattern compared with the other strains; however, at wavelengths >600 nm, all strains had similar scattering patterns but different scattering intensities (Figure 3b). Cross‐validation of PCA (Figure 5) and *k*NN classification shows 100% classification accuracy for all *Salmonella* strains (Table 2), indicating that each strain had distinguishable hyperspectral signatures and can be differentiated using the developed protocol with 100% accuracy.

**Figure 6:**
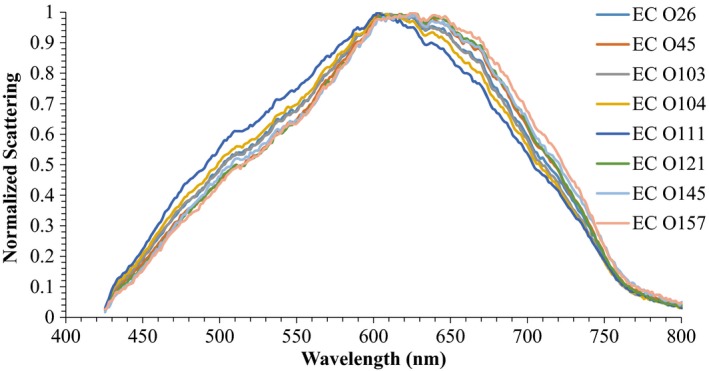
Hyperspectral graphs of *Escherichia coli* (EC) O26, O45, O103, O104, O111, O121, O145, and O157

**Figure 7:**
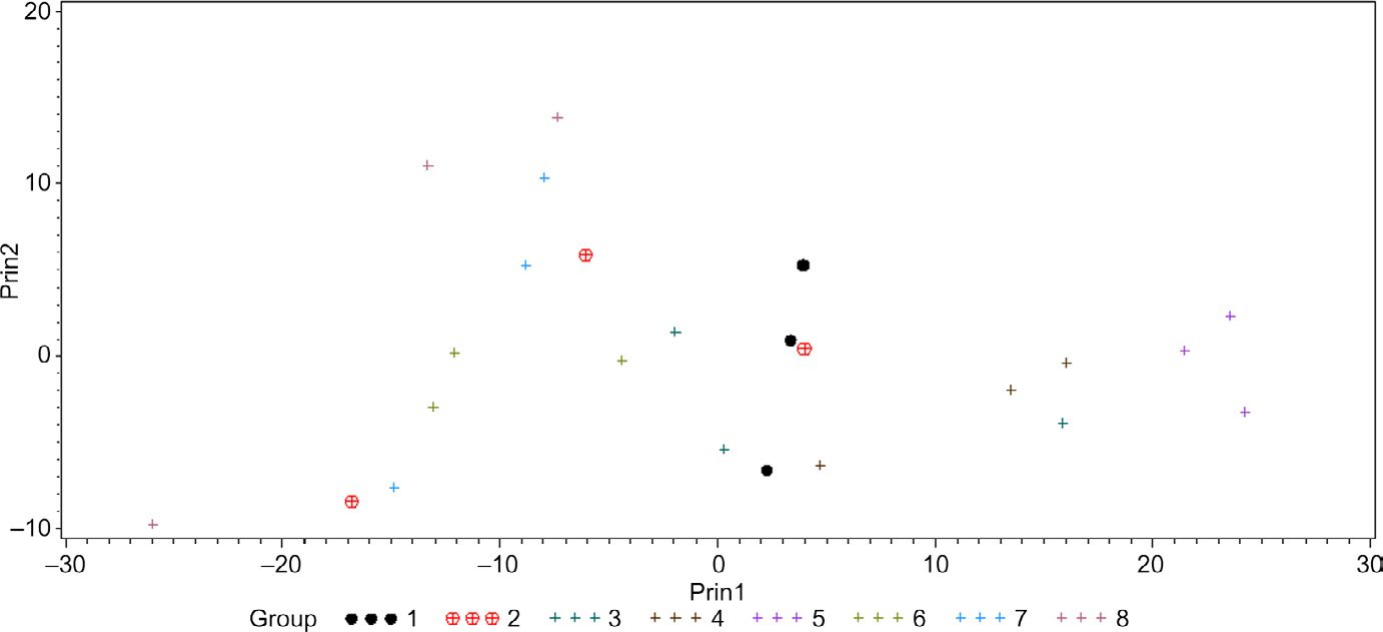
Principal component analysis (PCA) plot showing first two principal components (Prin1 and Prin2) of hyperspectral graphs of *Escherichia coli* O26 (1), O45 (2), O103 (3), O104 (4), O111 (5), O121 (6), O145 (7), and O157 (8)

Among *E. coli* serotypes, although all serotypes had a different intensity of scattering, they followed a similar pattern throughout the wavelength range (Figure 6). Approximate grouping of various serotypes of *E. coli* can be visualized from the PCA plot presented in Figure 7. Except *E. coli* O26, O45, and O121, which had classification accuracy of 66.67%, classification accuracy of all other *E. coli* serotypes was 100% (Table 2). *Escherichia coli* O26, O45, and O121 were misclassified as *E. coli* O103, O121, and O45, respectively, at 33.33%.

**Figure 8:**
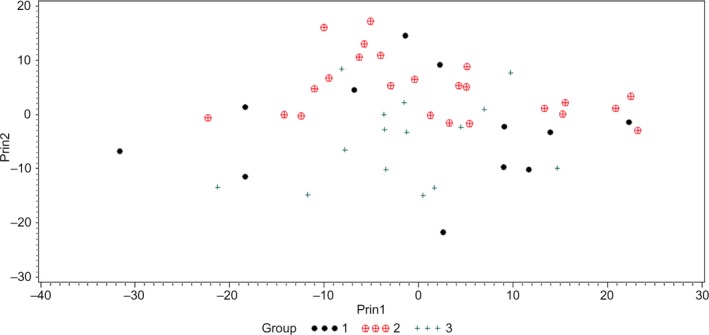
Principal component analysis (PCA) plot showing first two principal components (Prin1 and Prin2) of hyperspectral graphs of four strains of *Cronobacter sakazakii* (1); eight strains of *Escherichia coli* (2); and five strains of *Salmonella* spp. (3)

To study whether HSI could be used to differentiate various bacteria at the genus level, PCA (Figure 8) and *k*NN classification followed by cross‐validation was conducted using hyperspectra of various strains of *C. sakazakii*, *E. coli*, and *Salmonella* spp. Overall, low values of classification accuracy were obtained for *C. sakazakii* (58.33%) and *Salmonella* spp. (20%); however, *E. coli* had 79.17% classification accuracy. This poor classification accuracy could be explained from the results presented in Table 2. As each strain within a genus had distinguished hyperspectral signatures and was different from other strains in the same genus, it is difficult to group together various strains within a genus on the basis of *k* NN modeling technique used in this study.

**Figure 9:**
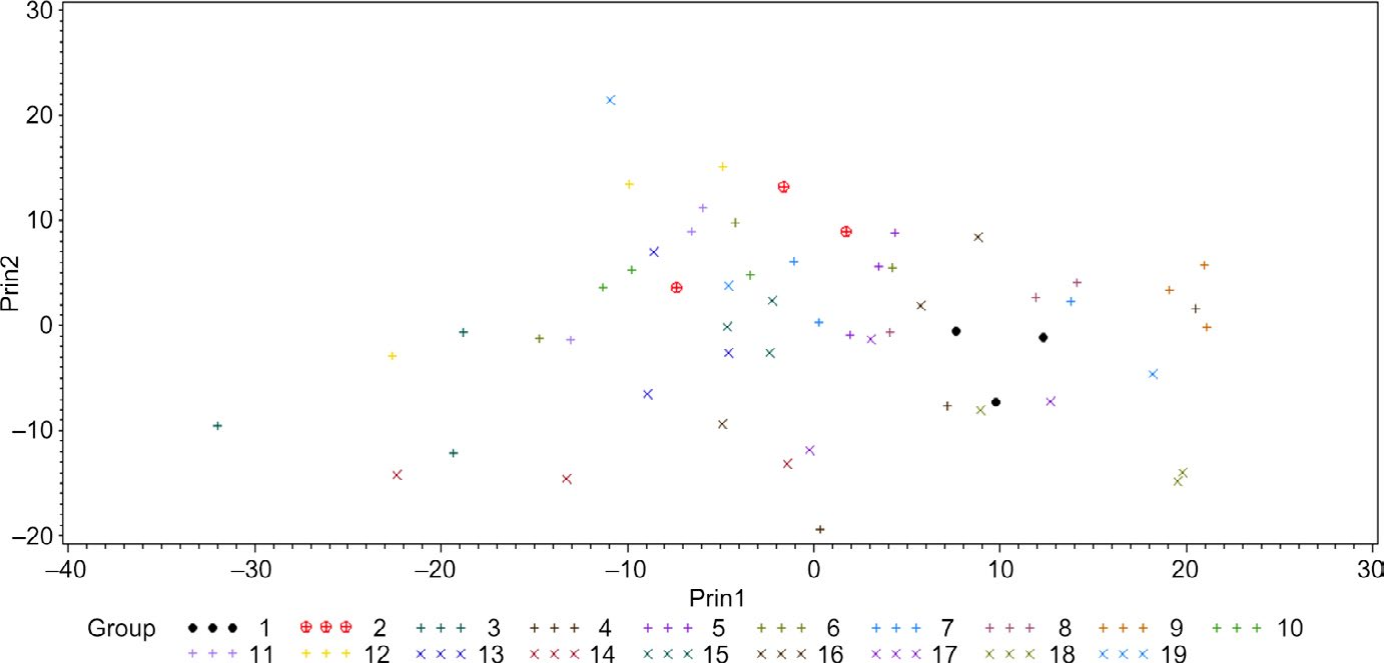
Principal component analysis (PCA) plot showing first two principal components (Prin1 and Prin2) of hyperspectral graphs of *Cronobacter sakazakii* BAA‐894 (1), E1 (2), P1 (3), and P2 (4); *Escherichia coli* O26 (5), O45 (6), O103 (7), O104 (8), O111 (9), O121 (10), O145 (11), and O157 (12); *Salmonella* Agona BAA‐707 (13), Montevideo (14), Senftenberg (15), Tennessee (16), and Typhimurium (17); *Listeria monocytogenes* (18); *and Staphylococcus aureus* (19)

To study the efficacy of HSI to differentiate bacteria when different strains from different genera are analyzed together, PCA (Figure 9) and *k*NN classification of hyperspectral graphs of various strains of *C*. *sakazakii*, *E. coli* and *Salmonella* spp., and *L. monocytogenes* and *S. aureus* was conducted. Cross‐validation accuracy of the classification of different strains is presented in Table 3. Overall, classification accuracy of these strains was not good. Only *C. sakazakii* P1, *S*. Montevideo, *E. coli* O104 and O111, and *L. monocytogenes* had 100% classification accuracy, whereas all other strains were classified at accuracy of ≤66.67%. *Salmonella* Tennessee and *E. coli* O45 were the most poorly classified strains with an accuracy of 0%. No specific differentiation among the Gram‐positive and Gram‐negative strains used in this study can be made from the PCA data presented in Figure 9. However, by including more strains of *L. monocytogenes, S. aureus*, and other Gram‐positive bacteria in future studies, the accuracy of HSI to differentiate Gram‐positive bacteria from Gram‐negative can be studied.

**Table 3:** Classification accuracy of various strains of *Cronobacter sakazakii*, *Salmonella* spp. and *Escherichia coli*, and *Listeria monocytogenes* and *Staphylococcus aureus* when analyzed together obtained from cross‐validation of principal component analysis and *k*NN (*k*‐nearest neighbor, *k* = 3) classification*

Bacteria	Strain/Serotype	% Classification Accuracy	Misclassified As (%)
*Cronobacter sakazakii*	BAA–894	33.33	CS‐P2 (33.33), EC‐O104 (33.33)
	E1	33.33	Others (66.67)
	P1	100	–
	P2	33.33	CS‐BAA−894 (33.33), Others (33.33)
*Salmonella* spp.	SA	33.33	EC‐O45 (33.33), EC‐O103 (33.33)
	SM	100	–
	SS	66.67	Others (33.33)
	ST	0	EC‐O26 (33.33), EC‐O121 (33.33), Others (33.33)
	STy	33.33	CS‐BAA–894 (66.67)
*Escherichia coli*	O26	66.667	ST (33.33)
	O45	0	EC‐O121 (33.33), Others (66.67)
	O103	66.67	ST (33.33)
	O104	100	–
	O111	100	–
	O121	66.67	EC‐O45 (33.33)
	O145	100	–
	O157	66.67	EC‐O121 (33.33)
*Listeria monocytogenes*	SLR 2249	100	ATCC
*Staphylococcus aureus*	25923	66.67	LM (33.33)

**Figure 10:**
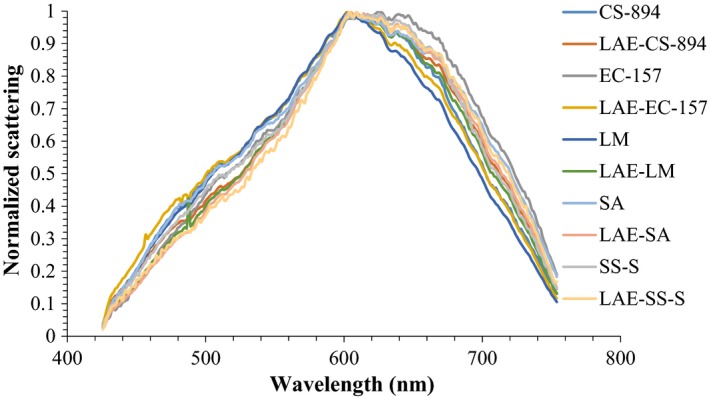
Hyperspectral graphs of lauric arginate (LAE)‐treated and nontreated *Cronobacter sakazakii* BAA‐984 (CS‐894), *Escherichia coli* O157 (EC‐157), *Listeria monocytogenes* SLR 2249 (LM), *Staphylococcus aureus* 25923 (SA), and *Salmonella* Senftenberg (SS‐S)

Becerril et al. (2013) reported that bacterial cell membranes were the main targets of LAE treatment, which resulted in the inactivation of cells by the disruption of cell membranes. Using a scanning electron microscope, they also demonstrated that cell membrane disruption resulted in structural changes in bacterial cells. No differences were observed between the hyperspectral images of LAE‐treated and nontreated bacterial cells of various strains at 4,000 × magnification. However, LAE treatment of various strains significantly affected their hyperspectral signatures. As seen in Figure 10, LAE‐treated strains had different intensities over the entire range of wavelengths compared with their corresponding nontreated strains. Classification accuracy of LAE‐treated and nontreated within each bacterium was 100%, therefore, suggesting that LAE‐treated bacterial cells can be differentiated from healthy and nontreated cells. No specific trend in the change of hyperspectra of various strains due to LAE treatment along the wavelengths was observed; however, overall differences in scattering intensities between LAE‐treated and nontreated strains were greater at >600nm.

Yoon et al. (2013b) used HSI over the wavelength of 400 to 1,000 nm (with 1.29 nm wavelength separation) for the identification and differentiation of pure culture STEC colonies grown on Rainbow agar at 37°C for 24 hr. They used PCA‐*MD* (Mahalanobis distance) and PCA‐*k*NN (*k* = 3) models for the classification prediction of STEC colonies and applied hold one out and *k*‐fold CV (cross‐validation) techniques for the validation of classification models. Yoon et al. (2013b) reported that *E. coli* O111 and O121 showed the highest classification accuracy of >99%, irrespective of classification modeling technique used, whereas *E. coli* O26, O45, O103, and O145 showed the accuracy between 84% and 100% depending on the preprocessing technique used.

In another study, Yoon et al. (2013a) used HSI (at 400 to 1,000 nm wavelength range) and various prediction models for classification of STEC colonies plated on Rainbow agar as mixed cultures. They concluded that the best prediction model was *k*NN classification when standard normal variate and detrending, and first derivative and spectral smoothing were used as preprocessing techniques. Using this *k*NN model, Yoon et al. (2013a) reported that classification accuracy of *E. coli* O26, O45, O103, O111, O121, and O145 was 95.80, 100, 88.54, 100, 100, and 91.93%, respectively. Yoon et al. (2013a) also concluded that hyperspectral graphs from wavelengths 750 to 1,000 nm did not provide important information for the classification and differentiation purposes of bacterial strains. These conclusions are in agreement with the current study's findings because hyperspectral graphs generated in this study at wavelengths ranging from ~750 to 1,000 nm for different bacterial strains were overlapping and indistinguishable, and could not be utilized for the classification purposes. Yoon et al. (2013a) also expressed a concern regarding morphology of bacterial colonies that could interfere with HSI and hence affecting hyperspectral signatures.

CytoViva^®^ conducted a study to identify and differentiate spores of *Bacillus globigii* and *B. anthracis* on the basis of their hyperspectral signatures. They reported that normalized hyperspectra of spores of two *Bacillus* strains had significantly different shapes and peaks (CytoVivo^®^, n.a.). However, in the current study, we observed very similar shaped hyperspectra and peaks of strains (but with different scattering intensities) within a same genus. These differences between CytoViva^®^ and our results could be attributed to the differences in the biochemical structure of spores and vegetative bacterial cells. Bacterial cells consist of basic cellular components common to most of the prokaryotes (such as cell membrane, cytoplasm, and DNA), whereas bacterial spores have entirely different structure consisting of different proteins (compared with cells) and seven different layers over the core of spores (Montville & Mathews, 2005).

Currently, preassembled and preprogrammed HSI systems, such as CytoViva^®^, are quite expensive and can easily cost over $125,000. This high initial investment cost is one of the major factors that restricts the research and application of HSI in the field of food microbiology. However, this initial cost can be reduced by mounting commercially available hyperspectral cameras on regular laboratory compound microscopes and using predeveloped imaging software (such as ENVI). At the current technology, this self‐assembly process can reduce the initial investment cost to less than one‐fifth of a preassembled HSI system. Once the HSI is installed, the cost of running HSI analysis is considerably low, which includes the cost of isolation media and regular microbiology laboratory tools (such as loops, glass slides, cover slips, and biosafety cabinet). In terms of analyzing time, once the bacterial colonies are isolated on an appropriate agar, the hyperspectral imaging acquiring and analyzing time is less than 15 min, which can be reduced further with more research and developing standard methods.

## 4 CONCLUSIONS

In conclusion, overall classification accuracy of bacterial strains used in this study was acceptable when *k*NN classification model and cross‐validation were applied within a specific genus. However, poor classification of various strains when compared together strongly suggests a need of further investigation, and use of different classification models and validation techniques, to eliminate the experimental and statistical factors responsible for poor classification. The HSI protocols should also be tested and validated for isolation and immobilization of bacterial cells from various food matrices. Although at the present state of the technology, HSI cannot replace the traditional microbiological identification methods, but it can be used effectively at presumptive levels for the identification of pathogens.

## CONFLICTS OF INTEREST

All authors declare no conflicts of interest in this article.

## ETHICAL STATEMENT

This study does not involve any human or mammal testing.
